# Limits of radiomic-based entropy as a surrogate of tumor heterogeneity: ROI-area, acquisition protocol and tissue site exert substantial influence

**DOI:** 10.1038/s41598-017-08310-5

**Published:** 2017-08-11

**Authors:** Laurent Dercle, Samy Ammari, Mathilde Bateson, Paul Blanc Durand, Eva Haspinger, Christophe Massard, Cyril Jaudet, Andrea Varga, Eric Deutsch, Jean-Charles Soria, Charles Ferté

**Affiliations:** 1 INSERM U1015, Equipe Labellisée Ligue Nationale Contre le Cancer, Gustave Roussy Cancer Campus, Villejuif, France; 2 Département de l′imagerie médicale, Gustave Roussy, Université Paris Saclay, F-94805 Villejuif, France; 3 Department of Radiology, Columbia University Medical Center, New York, New York, USA; 4 Département d’Innovation Thérapeutique et des Essais Précoces (DITEP), Gustave Roussy, Université Paris Saclay, F-94805 Villejuif, France; 5 Institut Hypercube, Paris, France; 6 Department of Radiotherapy, UZ Brussel, Brussels, Belgium; 7 Département de radiothérapie, Gustave Roussy Cancer Campus, Université Paris Saclay, F-94805 Villejuif, France; 8 INSERM U981, Biomarqueurs prédictifs et nouvelles stratégies en oncologie, Université Paris Sud, Gustave Roussy, Villejuif, France; 9 INSERM U1030, Paris Sud University, Gustave Roussy, Villejuif, France

## Abstract

Entropy is a promising quantitative imaging biomarker for characterizing cancer imaging phenotype. Entropy has been associated with tumor gene expression, tumor metabolism, tumor stage, patient prognosis, and treatment response. Our hypothesis states that tumor-specific biomarkers such as entropy should be correlated between synchronous metastases. Therefore, a significant proportion of the variance of entropy should be attributed to the malignant process. We analyzed 112 patients with matched/paired synchronous metastases (SM#1 and SM#2) prospectively enrolled in the MOSCATO-01 clinical trial. Imaging features were extracted from Regions Of Interest (ROI) delineated on CT-scan using TexRAD software. We showed that synchronous metastasis entropy was correlated across 5 Spatial Scale Filters: Spearman’s Rho ranged between 0.41 and 0.59 (P = 0.0001, Bonferroni correction). Multivariate linear analysis revealed that entropy in SM#1 is significantly associated with (i) primary tumor type; (ii) entropy in SM#2 (same malignant process); (iii) ROI area size; (iv) metastasis site; and (v) entropy in the psoas muscle (reference tissue). Entropy was a logarithmic function of ROI area in normal control tissues (aorta, psoas) and in mathematical models (P < 0.01). We concluded that entropy is a tumor-specific metric only if confounding factors are corrected.

## Introduction

Tumors exhibit an extensive genetic and phenotypic variation. This translates to a heterogeneity that can be observed at different scales: between patients, across metastases from the same primary tumor, or within a single metastasis. Importantly, intra and inter-tumor heterogeneity also undergoes temporal variation due to genomic instability and is recognized as a prominent factor leading to cancer treatment failure and poor prognosis^1–6^. Spatial and temporal heterogeneity can be accurately estimated through sophisticated genomic analyses, but these approaches typically require invasive tumor biopsies and are also limited to the sampling site^1–6^.

Recent progress in the medical image-computing field has allowed for the extraction of advanced quantitative imaging biomarkers without any additional examinations or costs. These noninvasive biomarkers describe the tumor’s imaging phenotype and extract metrics that measure *in vivo* the inner organization of any visible metastasis (i.e. virtual biopsies). They can evaluate the spatial dissemination of the malignant process as well as the surrounding tissue (i.e. host). Additionally, they can assess the temporal evolution of the malignancy since medical imaging procedures are performed regularly across the treatment sequence in most cancer patients (i.e. diagnosis, staging, and treatment monitoring). Medical imaging could thus measure the heterogeneity of the tumor imaging phenotype along the treatment sequence or course of disease and provide valuable new information.

**Figure 1:**
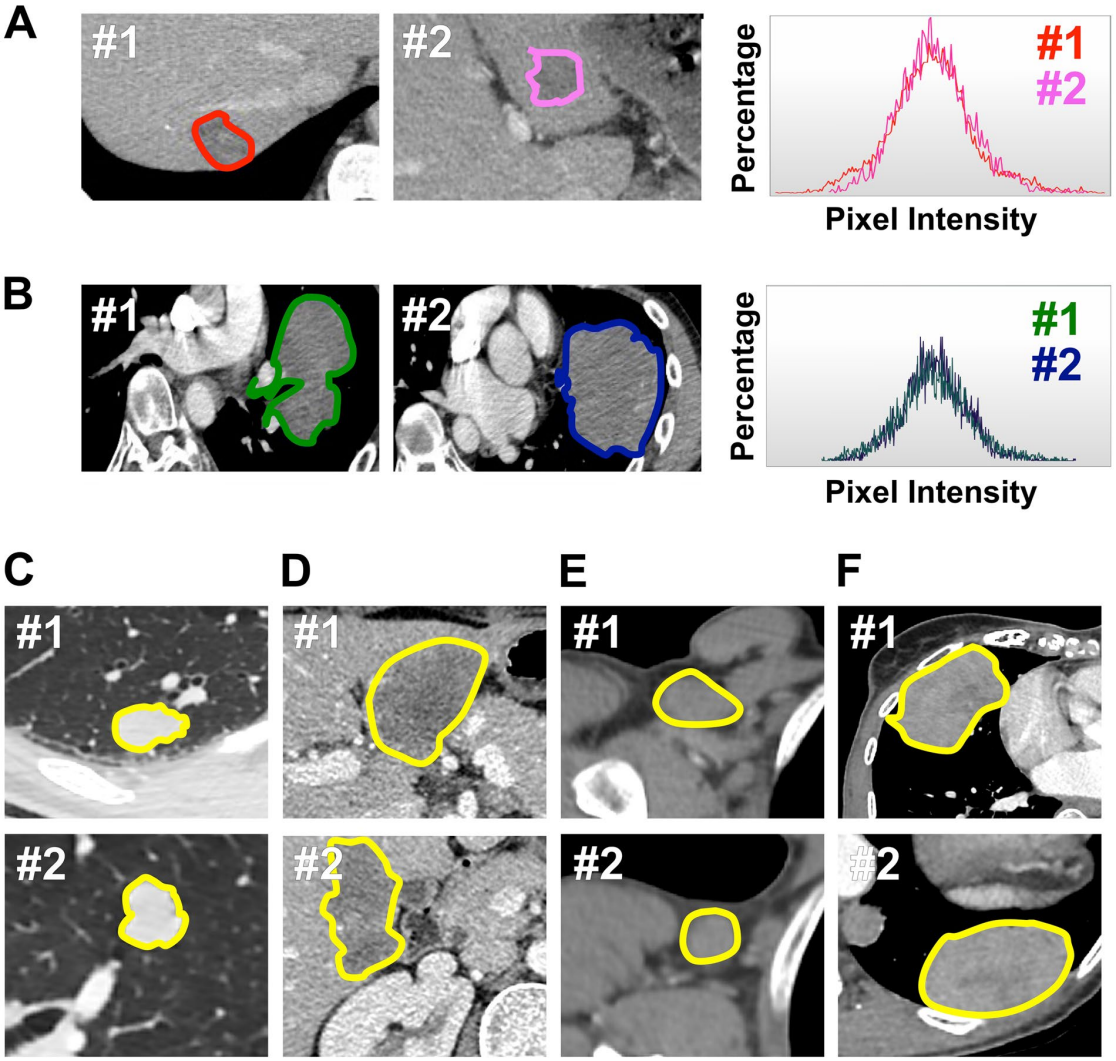
Comparing the imaging phenotype of paired biopsy-proven synchronous metastases. In each patient (n = 112), we compared the imaging phenotype of two biopsy-proven synchronous metastases (#1 vs. #2) from the same primary tumor and within the same organ. Patients (**A–F**) demonstrate the similarity of imaging phenotype between two synchronous metastases captured by trained radiologists (picture #1 and #2) and by TexRAD software (histogram #1 vs. #2).

First order statistics provide the most widely used metrics for the quantification of imaging phenotype. Imaging biomarkers are often extracted from a single two-dimensional CT-scan slice. The delineation of the entire three-dimensional tumor volume is indeed difficult to routinely perform in clinical practice. The research community has developed several ready-to-fit solutions such as TexRAD (Cambridge, United Kingdom), the key advantage of which is the apparent simplicity of the process. The tumor is segmented on CT-scan (an ROI or Region Of Interest is defined) and the signal intensity in each voxel is calculated. This corresponds to the attenuation of X-ray beam photons, which in turn is represented by the density in Hounsfield Units of the tissue (difference of density in comparison to water). Different spatial scale filters (SSFs) will ultimately modify all of these outputs (Fig. 1). Finally, the software computes Shannon’s entropy and other low order textural features. Beyond TexRAD, the radiomics community has developed a wide range of solutions allowing for the extraction of those metrics and an even larger dataset of imaging features through simple pipelines. The extraction is based on open-source and free solutions with inter-operable locked docker containers (e.g. Radiomics.io using 3D-Slicer^7^, IBEX^8^, LIFEX^9^) or home-grown robust algorithms^10^.

An increasing body of studies in the literature are proving the potential clinical value of Shannon’s entropy calculated after a Laplacian of Gaussian transformation using the settings included in the TexRAD software^11–19^. These studies have depicted that entropy is associated with staging^11, 12^, outcome^13–16^, expression of molecular pathways such as tumor metabolism^11, 12^, and treatment response^11–15, 17–19^ in esophageal, lung, colorectal, and head and neck cancers. These articles have received very positive feedback from the oncology and radiology communities, especially considering the critical need for alternative imaging biomarkers. This need has arisen with the advent of precision medicine and new drugs associated with atypical patterns of response that get misclassified by conventional response criteria^20–28^.

The quantification of spatial or temporal heterogeneity in the cancer imaging phenotype requires the identification of robust tumor-specific quantitative metrics. Recent studies in radiomics, however, have raised awareness about the importance of properly setting imaging acquisition parameters^10^. Indeed, any valid imaging biomarker changes should be attributed to changes in tumor biological characteristics and not to confounding factors. As entropy is one of the most promising metrics according to the current literature, we further explored its significance.

Our underlying assumption is that tumor-specific quantitative metrics can be identified by a comparison of paired synchronous metastases (SM) issued from the same primary tumor, developing at the same time and in the same organ of a given patient. These SM are indeed expected to share important phenotypic similarities (genomic and radiomic), as they are seeds spreading at the same time and in the same soil^29^. Our hypothesis states that entropy should be correlated between matched and paired biopsy-proven SM, and that a significant proportion of the variance of entropy should be attributed to the malignant process and not to confounding factors. To this end, we used a large series of patients prospectively enrolled in the MOSCATO-01 precision medicine trial.

## Results

### Patient characteristics

We screened a cohort of 525 pts. The most frequent primary tumor types were lung (n = 102 pts, 19%), head and neck (n = 65 pts, 12%), colorectal (n = 64 pts, 12%) and urothelium (n = 99 pts, 19%).

We included 112 pts with paired synchronous biopsy proven metastases (SM#1 and SM#2) in a single organ. The most frequent primary tumor types in this group were lung (n = 28 pts, 25%), head and neck (n = 15 pts, 13%), colorectal (n = 14 pts, 12%), urothelium (n = 7 pts, 6%), prostate (n = 7 pts, 6%), and liver and bile duct (n = 6 pts, 5%). The sites of the biopsies were lung (n = 36 pts, 32%), liver (n = 35 pts, 31%), lymph nodes (n = 25 pts, 22%) and viscera (n = 16 pts, 14%).

### Lesion characteristics

The average ± standard deviation of entropy in the 112 patients was 4.36 ± 0.62 in SM#1, 4.15 ± 0.85 in SM#2, 4.09 ± 0.53 in the psoas muscle, and 4.42 ± 0.52 in the aorta. The average ± standard deviation of ROI area (pixels) in the 112 patients was 978 ± 1727 in SM#1, 723 ± 1510 in SM#2, 452 ± 350 in the psoas muscle and 1492 ± 1210 in the aorta.

### Comparison of the imaging phenotype of synchronous metastases

**Figure 2:**
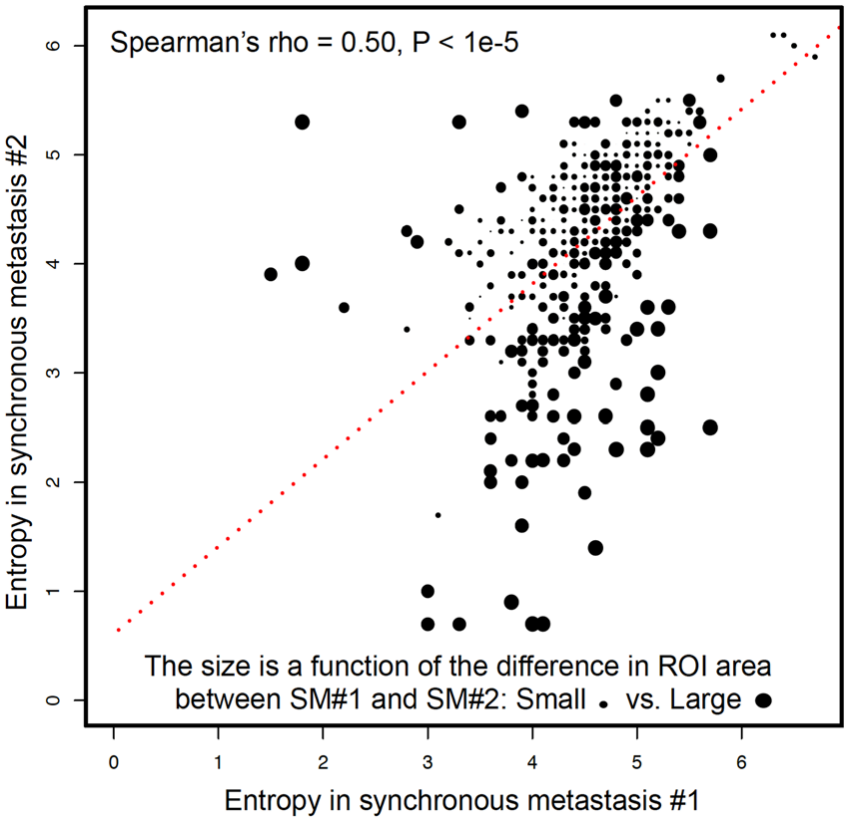
Comparing Entropy in paired synchronous metastases. Entropy is correlated between paired synchronous metastases across all SSFs (SM#1 vs. SM#2). We observed that larger differences in entropy were explained by larger differences in ROI area.

We explored the similarity of imaging phenotype between 112 pairs of biopsy-proven synchronous metastases occurring at the same time and in the same organ of a given patient (Fig. 1). To this end, we computed the Spearman’s rho correlation coefficient between entropy in SM#1 vs. entropy in SM#2. The Spearman’s rho was 0.59-0.57-0.55-0.41-0.41 at spatial scale filters (SSF) 2-3-4-5-6, respectively (P = 0.0001, Bonferroni correction, Fig. 2).

The Spearman’s rho coefficient between SM#1 vs. SM#2 (n = 112 pairs) was calculated for Mean (r = 0.68*), Standard Deviation (r = 0.43*), Mean Positive Pixels (r = 0.43*), Skewness (r = 0.24) and Kurtosis (r = −0.02). Some correlations were statistically significant after Bonferonni correction (*), however none of them outperformed entropy. The intra and inter-tumor (#1 vs. #1, #2 vs. #2) Spearman’s rho correlation coefficients are displayed as supplemental material.

### Multivariate linear analysis

We explored the proportion of the variance of entropy attributable to the malignant process as opposed to confounding factors. To this end, we fitted a multivariate linear analysis. Entropy in SM#1 was significantly associated with (Table 1): (i) the primary tumor type (e.g. lung adenocarcinoma, colorectal adenocarcinoma, head and neck squamous cell carcinoma, etc), (ii) the entropy in SM#2 (malignant pattern recognition), (iii) the SM#1 ROI area, (iv) the anatomical metastasis site (e.g. liver, lymph node, lung, etc), and (v) the entropy in the psoas muscle (normal control tissue). We obtained the same results at each SSF (2-3-4-5-6) independently. Table 1 pools all of the SSFs. Consequently, the primary tumor type and the malignant process explain a significant portion of the variance of entropy but there are confounding variables that alter the output.

**Table 1:** Multivariate linear analysis of the increase of entropy in synchronous metastases #1.

Variable	Coefficient (estimate)	Significance (P Value)
Intercept (reference)	1.96	<10^−5^
Entropy of synchronous metastasis #2 (malignant process)	0.06	<10^−3^
ROI area of synchronous metastasis #1 (volume-dependence)	0.65	<10^−5^
Entropy of psoas muscle (acquisition protocol)	0.15	<10^−5^
Site of the metastasis		
Lymph node		
Liver	0.12	0.03
Lung	−0.08	0.17
Viscera	0.14	0.03
Primary Tumor		
Prostate		
Urothelium	−0.15	0.08
Lung or pleura	−0.20	0.02
Digestive tract	−0.04	0.68
Liver and bile ducts	0.07	0.50
ENT and thymus	−0.22	0.01
Ovary, Uterus, Testicle	-0.11	0.22
Breast	−0.15	0.19
Pancreas	−0.23	0.03
Other	−0.21	0.03

Multivariate linear analysis shows that entropy in SM#1 is a function of the ROI area of SM#1 (volume-dependence), the entropy of SM#2 (malignant pattern recognition), the entropy of the psoas muscle (acquisition protocol), the site of metastasis, and the primary tumor.

### Association between ROI area and entropy

Since the ROI area size explains a significant portion of the variance of entropy, we further explored the association between the entropy and the ROI area in three different settings: (i) malignant tissues, (ii) normal tissues and (iii) mathematical models.

**Figure 3:**
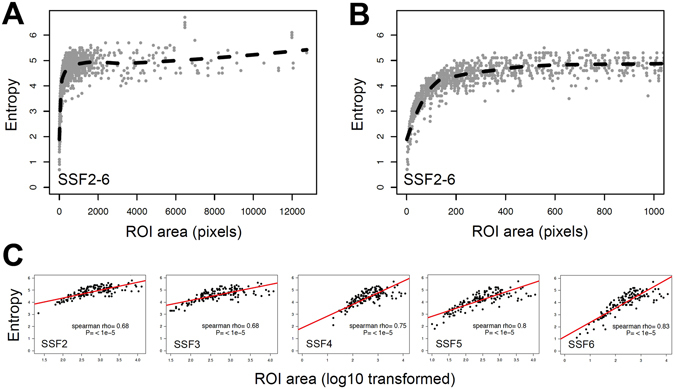
Entropy is a logarithmic function of the area of the region of interest. (**A**,**B** and **C**) are graphs showing the entropy across all SSFs (**A**: ROI area from 0 to 12,000 pixels, **B**: zoom on ROI area smaller than 1,000 pixels) and for all SSF independently (**C**) in function of the area of the ROI in pixels within synchronous metastases #1. We observed that in every type of tissue (metastasis, normal psoas muscle, and blood in the aorta), entropy is a logarithmic function of the area of the ROI (P < 0.01, R2 = 0.47) rather than a linear one (P < 0.01, R2 = 0.14).

First, we computed the association in the entire set of synchronous metastases (n = 224 lesions). We observed that entropy was directly correlated with ROI area when the ROI was smaller than 200 pixels and reached a plateau when the ROI was larger than 200 pixels (Fig. 3). There was a strong linear association between log10 (ROI area in pixels) and entropy: spearman rho = 0.8 (P < 1e-5).

Second, we explored the association in normal control tissues (i.e. non-malignant tissues). We showed the same strong association of ROI area and entropy in the psoas muscle (P < 0.001) and the aorta (P < 0.001).

**Figure 4:**
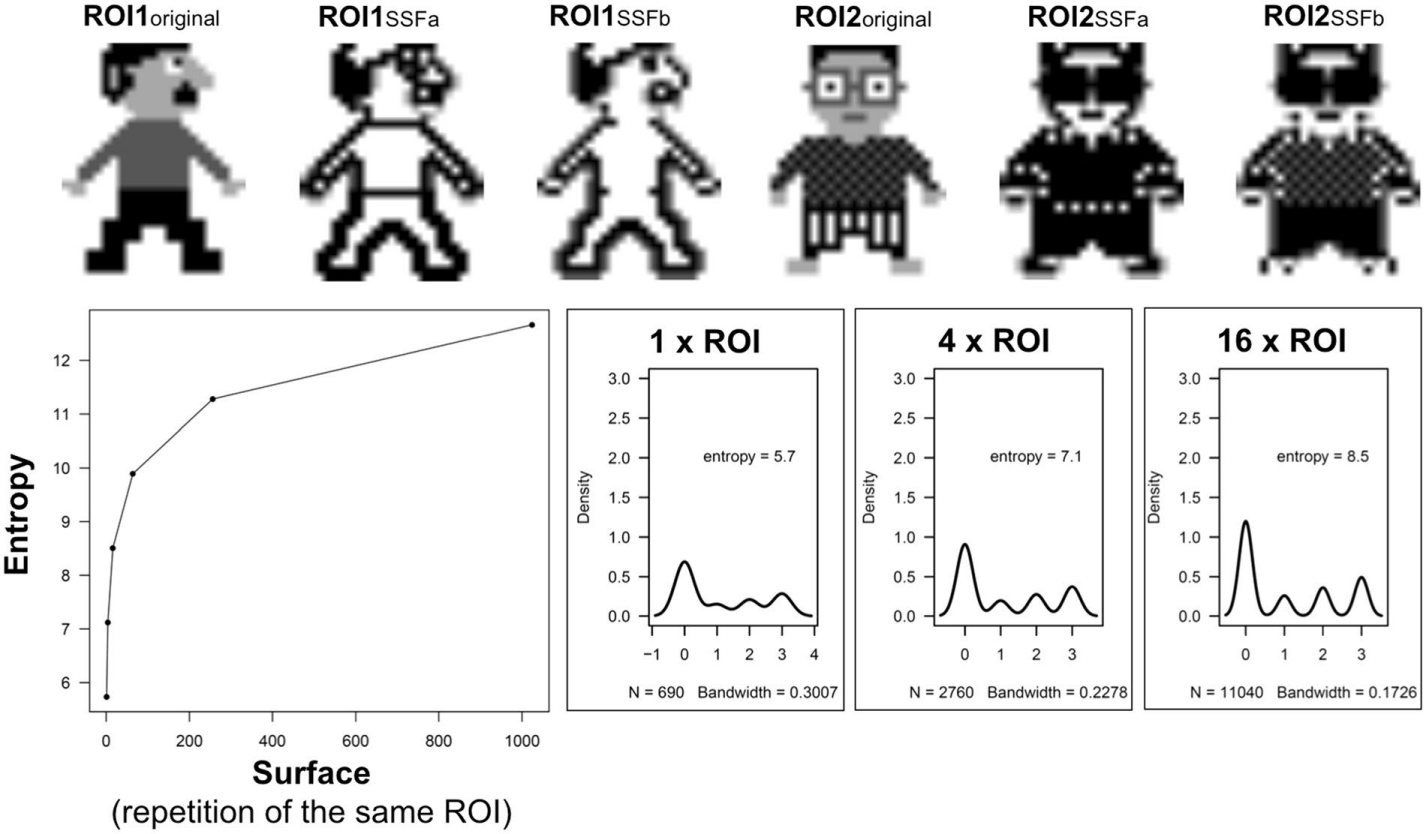
Mathematical model evaluating the evolution of Entropy in function of the surface of the ROI and the impact of SSF. We calculated the entropy in function of the surface of the ROI. We defined the surface by the number of repetitions of the same 690 pixel ROI (i.e. ROI1_original_ and ROI2_original_). ROI1_original_ and ROI2_original_ have the same distribution of pixel intensity coded 0, 1, 2, and 3 and are associated with a corresponding white - grey - black signal intensity. It shows that (i) entropy is identical in very different ROIs, and (ii) entropy calculated after Laplacian of Gaussian filtering (SSFa and b such as used by TexRAD) remains area dependent although it captures a different pixel spatial distribution between ROI1 and ROI2.

Third, we calculated Shannon’s entropy in function of the number of repetitions of the same 690 pixel image (Fig. 4). The same trend was observed.

Consequently, the strong association between the entropy and the ROI area was demonstrated in all models, whichever tissue was considered.

### Association between ROI area and entropy in SM#1 vs. SM#2

We investigated if the difference in entropy between SM could be explained by the difference in tumor ROI area. We thus computed the distribution of entropy in SM#1 and SM#2 in function of the ROI area (Figs 3 and 4). The inter-tumor difference in entropy appeared to be a linear function of inter-tumor difference in ROI area (P < 0.01). We observed that tumors exhibiting the higher correlations in terms of entropy were also those with the most similar ROI area (Fig. 3).

### Normal control tissue

We explored whether the entropy from SM#1 was correlated with the entropy in a normal control tissue: the psoas muscle. We observed a significant correlation: Spearman Rho = 0.41 (P < 1e-5).

### Prognostic value of entropy for overall survival

Since entropy was similar between synchronous metastases and could be a tumor-specific imaging biomarker, we computed the prognostic value for overall survival analysis. To this end, we calculated the association between entropy within biopsy-proven SM#1 and overall survival in the entire cohort of screened patients (n = 525 pts).

**Figure 5:**
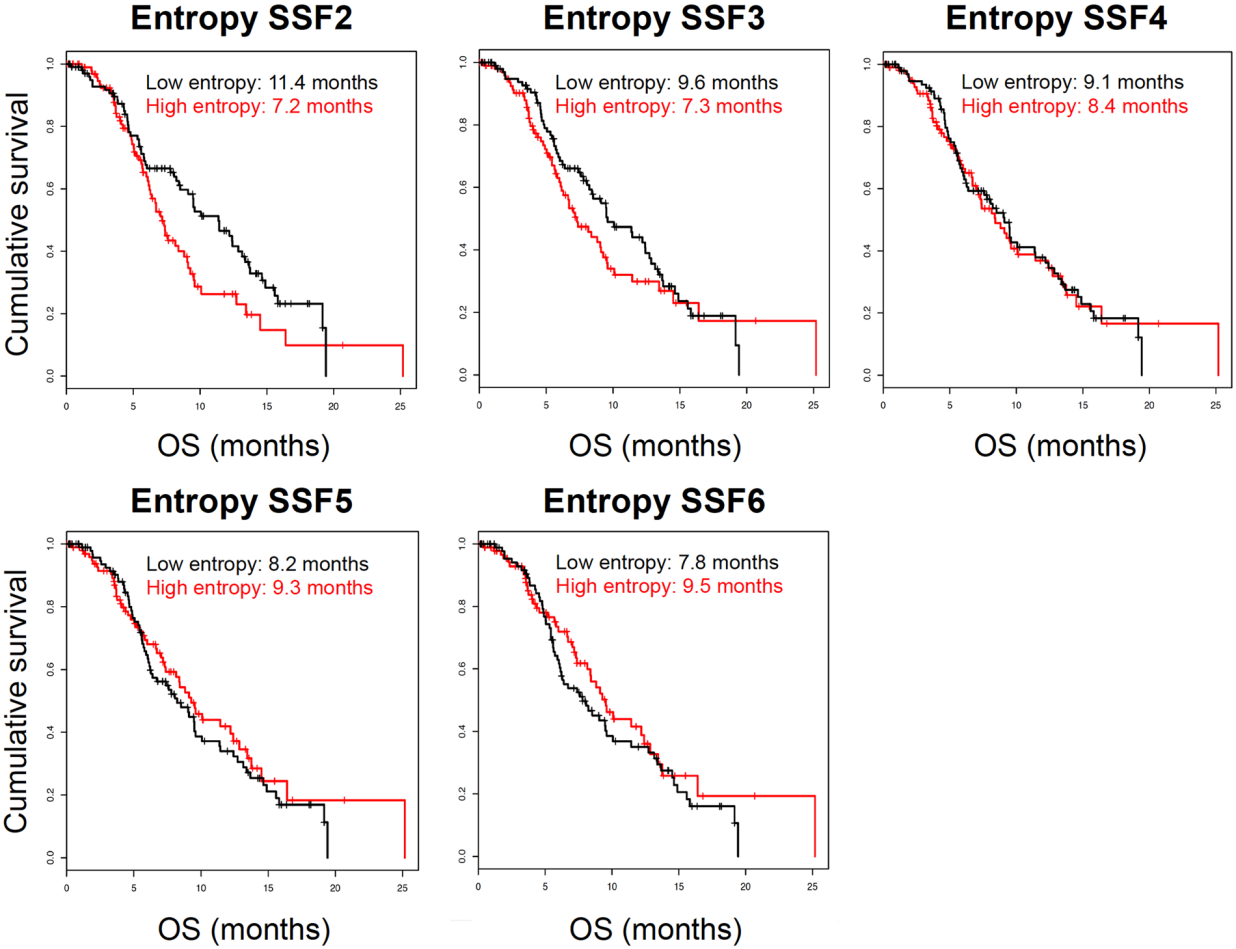
Association between metastases’ entropy and patients’ overall survival. Kaplan Meier estimates show the cumulative overall survival in patients with low and high entropy (sample median entropy was used to define the high- and low- groups) according to different spatial scale filters (SSF2-6). The association between high entropy and OS changes across SSFs: this illustrates the problem of false positives due to type I error and publication bias retaining only positive results.

The comparison between groups could not be analyzed with the Kaplan Meier method because the curves crossed each other many times across SSFs, therefore violating the condition for use of the log-rank test (Fig. 5). Strikingly, the sign of the associations tended to reverse from SSF2 (High entropy = 7.17 months, Low entropy = 11.40 months) to SSF6 (High entropy = 9.53 months, Low entropy = 7.79 months). Consequently, the effect of SSF is substantial, and influences the association between entropy and survival.

The lower and upper estimation of concordance index were 0.39 and 0.53 respectively. The concordance index was not significant (P-value > 0.19) which suggests that entropy does not predict overall survival.

## Discussion

Entropy has received special attention from the medical imaging community^9, 11–19, 31–34^. Our study proposed a methodology for exploration of the biological meaning of the entropy imaging feature as measured on clinical CT-scans. We demonstrated that entropy is specific to a given malignant process, but is also influenced by confounding factors.

We compared tumors sharing important biological similarities: paired biopsy-proven synchronous metastases. Synchronous metastases are indeed issued from the same primary tumor and are defined by simultaneous development in the cancer history of a given patient^29^. We compared the imaging phenotype of paired synchronous metastases occurring in the same organ as viewed by single time point CT-scan acquisitions. We showed that similar biological characteristics translated to similar entropy. By pooling data from multiple tumor types and sites, we showed that entropy is a function of the primary tumor type, the site of tumor growth (lymph node, lung, liver), and the specific malignant process (all P < .001). The complete comparison of paired synchronous metastasis imaging phenotypes and the stability of the imaging biomarkers across SSFs are shown in the supplemental materials (Supplemental Figs 1–2 and Supplemental Table 1–3).

The current mainstream hypothesis is that high entropy is instrumental to the appraisal of intra and inter-tumor heterogeneity through the radiomic approach. However, the present study sheds light on the potential caveats and biases in such a claim. We indeed found a significant impact from multiple confounding factors such as the ROI area size, the acquisition protocol, and the anatomical site of metastasis (Table 1). Taking these confounding variables into account is crucial if we are to revisit previously published results and robustly translate radiomic analysis into the clinical setting.

ROI area size is the most significant confounding factor that we need to take into account when computing Shannon entropy on CT-scans. According to our results, a proper measurement of the entropy within a tumor requires a minimum ROI area of 200 pixels in all tissue types (tumor, psoas muscle, aorta). The 200 pixel threshold should be applied prior to calculation of the entropy feature. This strong area-dependence constitutes a major technical challenge and caveat that might be partially explained by the finite sample size effect^35^. The pixel intensity distribution is indeed a continuous variable that is transformed into the probability of a discrete state: the continuous original intensity values are replaced by a single value representative of an interval (M = number of bins used for signal processing). This discretization leads to an expected systematic underestimation of entropy (because statistical fluctuations tend to decrease the distribution uniformity), that is only approximately calculable^36–38^: True entropy = observed entropy + (M−1)/2 N with N being the number of pixels analyzed within the ROI. In our experiment, the number of bins was the same in all patients but the number of pixels within ROIs was variable and thus smaller ROIs are expected to have greater underestimation. For example, the mean number of pixels within SM#1 was 978 leading to a theoretical underestimation of entropy from 3% to 13% if the bins are 64 and 256, respectively.

The demonstration of entropy’s dependence on logarithmic area is crucial^13^ because it may have biased the previous associations of entropy with: (i) staging, (ii) outcome, (iii) pathology, and (iv) treatment response^11–19, 31, 32^. First, entropy has been associated with clinical T-stage^11, 12^ but T-stage is defined by the size of the primary tumor. Second, entropy has predicted patient survival^13–16^ but tumor burden is a predictor of outcome^39^. Third, entropy was correlated with tumor glycolytic metabolism^11, 12^ but partial volume effect on PET is associated with lesion volume. Finally, in a wide range of cancer types^11–15, 17–19, 31, 32^, entropy is correlated with treatment response that is defined by variation in tumor diameter. Therefore, further studies will have to tackle this area/volume-dependence problem by investigating solutions such as sampling ROIs with a standardized area, defining a new variable such as an area-corrected entropy, or by reporting *a posteriori* the expected error bars.

Tumor heterogeneity is associated with poor prognosis and resistance to anticancer treatment across various tumor types^1–6^ and could be instrumental in predicting the efficacy of treatment with atypical patterns of response^24, 40–42^. We therefore explored the association between entropy and overall survival^6^. We showed the strong impact of signal processing (i.e. spatial scale filters) on entropy-outcome association in a large series of 525 patients. Since the SSFs (i.e. Laplacian of Gaussian transformation or spatial scale filters) are looking at different size features of the tumor (1–2 mm for fine and 5–6 mm for coarse), these conflicting results could be explained by the fact that they are evaluating different features or processes within the tumor. This needs to be explored and is beyond the scope of our study.

We showed that the entropy in a normal control tissue (psoas muscle) was associated with the entropy within the metastases. Although a full explanation of this association is beyond the scope of this paper, the inherent variability in image acquisition could explain this association. First, the intensity of the signal within voxels might be influenced by slight variations in the acquisition protocol. Second, the Laplacian of Gaussian kernel can be sensitive to the image noise and therefore affect the edge detection by the filter. Third, the contrast enhancement of the psoas muscle tissue and the metastases on CT-scan is subject to slight variability due to contrast enhancement product injection variability (increased volume, output, concentration) and to individual patient characteristics (decreased patient weight or cardiac output). The final hypothesis is that there is an association between the metastatic process and the skeletal muscle index^39^. Of note, it is difficult to find a perfect normal control tissue in sick and deconditioned cancer patients, especially since previous chemotoxic therapy has the potential to alter all types of tissue.

The caveat of Shannon’s entropy (used in TexRAD) should not be mistaken as a caveat of entropy itself. There are different models for the estimation of entropy and while Shannon’s is the most popular model, it is also the earliest and simplest model^36, 43^. Shannon’s entropy indeed systematically considerably overestimates entropy^35^ due to the erroneous underlying assumption that all pixels are independent (from their neighbor) and identically distributed. Consequently, a simple refinement could involve its measure at different scales of observation.

Similarly, the caveat of entropy should not be mistaken as caveat of Radiomics. Shannon’s entropy is indeed a first order statistic based only on the image histogram, which collapses the information regarding the spatial organization of voxels in the image to one dimension. As a comparison, second order features could be referred to as textural features because they investigate the spatial relationships between voxels such as distance, size zone matrix, and run length. Alternatives should be brought in for comparison in future imaging heterogeneity quantification: (i) Markov Random Fields are models emphasizing the dependencies between neighboring pixels^44, 45^ but face inherent computational complexity; (ii) simple and promising metrics are emerging from the field of geographical analysis^43, 46^ and allow for computation of the spatial configuration of pixels; (iii) the very active fields of Deep Learning and Deep Neural Networks are becoming increasingly efficient for tasks such as object and/or pattern recognition^47–50^.

Finally, our methodology could ultimately allow for the identification of new tumor-specific quantitative imaging metrics. The potential for identification of a radiomic signature specific to a given malignant process creates enticing research perspectives for virtual biopsy by imaging^16, 51–53^: (i) quantification of intra/inter-patient intra/inter-tumor spatial and temporal heterogeneity of the cancer imaging phenotype; (ii) deciphering the tumor microenvironment; (iii) computer-aided prediction of treatment efficacy or diagnosis of a malignant process.

In conclusion, the broad communities of radiology, oncology, and radiotherapy should be aware of the need to take into account the effects of ROI area size, metastatic site, and the individual characteristics of image acquisitions when quantifying and interpreting radiomic-based entropy as a tumor-specific surrogate for intra and inter-tumor heterogeneity. Only the proper evaluation and rigorous testing of nascent radiomic-biomarkers will allow their final implementation into the clinic.

## Methods

### Patients

We screened 525 consecutive patients prospectively enrolled in the MOSCATO-01 precision medicine trial (Gustave Roussy, Villejuif, France, NCT01566019). Patients were included after informed consent. The experimental protocol of the MOSCATO trial was carried out in accordance with guidelines and regulations and was approved by our institutional review board (Gustave Roussy, Villejuif, France). In this trial, each patient had a targeted tumor biopsy (synchronous metastases SM#1) to determine its molecular profile and was subsequently prescribed a molecular targeted therapy adapted to the molecular profile. The sample size was derived from power calculation, which is provided as a supplementary material.

### Synchronous metastases

Senior radiologists selected 112 patients out of 525 patients screened in MOSCATO trial presenting synchronous metastases (SM). Inclusion criteria were: (i) SM are within the same organ during the same acquisition; (ii) SM developed at the same time in the cancer history of a given patient; (iii) SM showed similarity in imaging phenotype according to visual analysis; (iv) SM have a minimal diameter of 1 cm (short axis). The main focus of this methodology is ensuring identical CT acquisition characteristics to minimize acquisition parameters as a source of intra-patient variability. To this end, using single acquisitions also ensures that: (i) the same contrast agent was injected at the same volume and rate; (ii) the contrast enhancement was identical (acquisition time was at the portal venous phase).

### Image acquisition

Whole body CT-scan acquisitions were obtained using a 64 HiSpeed spiral scanner (GE Medical Systems, Milwaukee, WI) after monophasic injection of monoionic contrast agent (Xenetix® 300; Guerbet, France). The typical CT parameters were: smooth convolution Kernel, 2 mm slice thickness, 1.4 mm slice interval, 0.7 s exposure time per rotation, tube current of 225 mAs, and 120 kVP.

### Radiomic feature extraction

TexRAD allows for the extraction of imaging features within a region of interest (ROI). Accordingly, four ROIs were delineated by senior radiologists in each patient: the largest cross section of the lesion considered for biopsy by the radiologist (SM#1), the largest cross section of the next largest synchronous metastasis in that same organ (SM#2), an ROI within the psoas muscle (delineated at the level of vertebra L3), and an ROI in the thoracic descending aorta. The first step was a filtering of signal intensity within the ROI, which defines the level and windows (minimum and maximum) in Hounsfield Units that would be considered for histogram analysis. Identical TexRAD filters designed for the analysis of soft tissue with contrast enhancement were used for the paired synchronous metastases (min: + 40HU: max: + 400HU). The second step was a filtering technique using a Laplacian of Gaussian band-pass filter. Five spatial scale filters (SSF2 to SSF6) evaluated the ROI at different scales with object radii of different sizes (2, 3, 4, 5, 6 mm) which are not dependent (invariant) on the pixel size (SSF2: 2 mm in radius to SSF6: 6 mm in radius) thus allowing evaluation of different imaging features (from fine to coarse features).

Entropy was defined as previously described^54^ by the equation: ${\rm{e}}=-\sum _{I=1}^{k}p(I)\ast log2(p(I))$ with k indicates grey-level from 1 to k, I reflects the intensity of the pixel value and p(I) the probability of the occurrence of that pixel intensity value. To note, in the literature this formula is also known as the Shannon entropy. It is considered, according to information theory, as a statistical measure of randomness and of the homogeneity of the number of voxels per level that corresponded to the amount of information that is needed to specify the full microstate of the system.

### Statistical methods

Descriptive statistics were performed using conventional metrics (mean, median, range). First, we compared the imaging phenotype of synchronous metastases by Spearman’s rho correlation coefficient with P-value corrected for multiple tests. Second, we evaluated if there was an association between confounding factors and entropy in SM#1 by multivariate linear regression model. Third, we evaluated the influence of the ROI area on the estimation of entropy by univariate regression model and non-parametric Spearman test. Fourth, we computed the difference in entropy between synchronous metastases as a function of the difference in ROI area by univariate regression model. Fifth, we explored whether the entropy from the SM#1 was associated with the entropy assessed in a ROI delineated in a reference non-tumoral tissue by univariate regression model and non-parametric Spearman test. Finally, we computed the prognostic value of entropy for overall survival analysis. We compared the overall survival in two groups: high entropy when entropy is above the median, and low entropy otherwise. Overall survival medians within these groups were estimated with the Kaplan Meier method. We computed the concordance index for validating the predictive ability of a model based on the increase in entropy for the prediction of overall survival. For this we used the package survcomp from Bioconductor^30^. Statistical analyses were performed using R version 3.3.0 and SPSS 24.0.

### Data Availability

The datasets generated and analyzed during the current study are available from the corresponding author.

## Electronic supplementary material

Supplementary Material Falanga et al
