# Supplementary material for: Limits of radiomic-based entropy as a surrogate of tumor heterogeneity: ROI-area, acquisition protocol and tissue site exert substantial influence
Source: Sci Rep. 2017 Aug 11;7:7952. doi: 10.1038/s41598-017-08310-5 (PMC5554130; doi:10.1038/s41598-017-08310-5)
Supplement: Supplementary file 1 — Supplementary Material Falanga et al [file 41598_2017_8310_MOESM1_ESM.pdf]

Title

Limits of radiomic-based entropy as a surrogate of tumor heterogeneity: ROI-area, acquisition protocol and tissue site exert substantial influence

Author list and affiliations

Laurent Dercle, MD, PhDc[1, 2, 3]; Samy Ammari, MD[2, 4]; Mathilde Bateson, MS[5]; Paul Blanc Durand, MD[4]; Eva Haspinger[4]; Christophe Massard, MD, PhD[4]; Cyril Jaudet, PhD[6]; Andrea Varga, MD[4]; Eric Deutsch, MD, PhD[7,8]; Jean-Charles Soria, MD, PhD[4, 8, 9]; Charles Ferté, MD, PhD[4, 8, 9]

1.INSERM U1015, Equipe Labellisée Ligue Nationale Contre le Cancer, Gustave Roussy Cancer Campus, Villejuif, France.

2. Département de l'imagerie médicale, Gustave Roussy, Université Paris Saclay, F-94805, VILLEJUIF France

3. Department of Radiology, Columbia University Medical Center, New York, New York, USA

4. Département d'Innovation Thérapeutique et des Essais Précoces (DITEP), Gustave Roussy, Université Paris Saclay, F-94805, VILLEJUIF France

5. Institut Hypercube, Paris, France

6. Department of Radiotherapy, UZ Brussel, Brussels, Belgium

7. Département de radiothérapie, Gustave Roussy Cancer Campus, Université Paris Saclay, F-94805, VILLEJUIF France

Supplemental Figure 1. Similarity of the imaging phenotype of 112 pairs of biopsy-proven synchronous metastases: inter and intra-tumor correlation

Figure 2 legend: Paired biopsy-proven synchronous metastases have a similar imaging phenotype as proved by the inter-tumor (#1 vs. #2) and intra-tumor (#1 vs. #1, #2 vs. #2) Spearman's rho correlation coefficient at various spatial scale filters (SSF2-6). Interestingly, we discovered a significant intra and inter-tumor correlation between entropy and the area of the ROI.

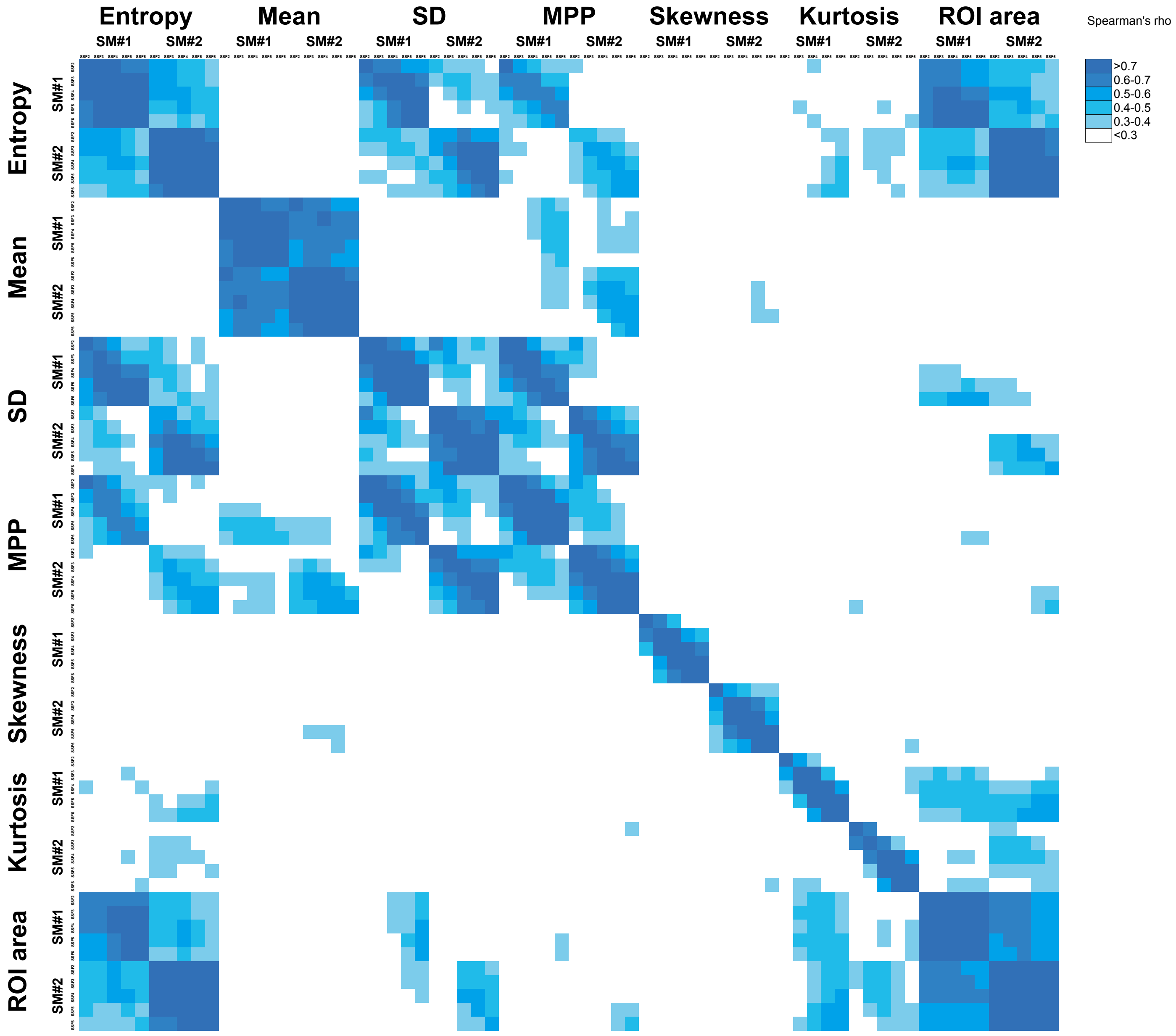

Supplemental Figure 2. Meaning of voxel intensity histogram analysis

Histogram analysis evaluates the number of voxels within the ROI (y-axis) for each tissue density measured in Hounsfield unit by Photon X attenuation (x-axis).

A. Shannon's entropy considers that all pixels are independent (from its neighbor) and identically distributed and thus does not perceive the heterogeneity of voxels spatial distribution. It explains that the picture at the left and the middle have the same histogram and thus the same entropy.

B. Entropy is considered as a surrogate of the heterogeneity of voxels intensity distribution.

C. Texrad extracts 6 features: Mean (average level of intensity), Mean Positive Pixel, Standard Deviation (SD, dispersion of intensity around the mean), Skewness (asymmetry of the histogram: is zero if the histogram is symmetrical around the mean, and is otherwise either positive or negative depending whether it is skewed above or below the mean), Kurtosis (peakedness, normalized to zero for a Gaussian-shaped histogram), Entropy (uniformity or randomness) and Total (number of pixels of the ROI).

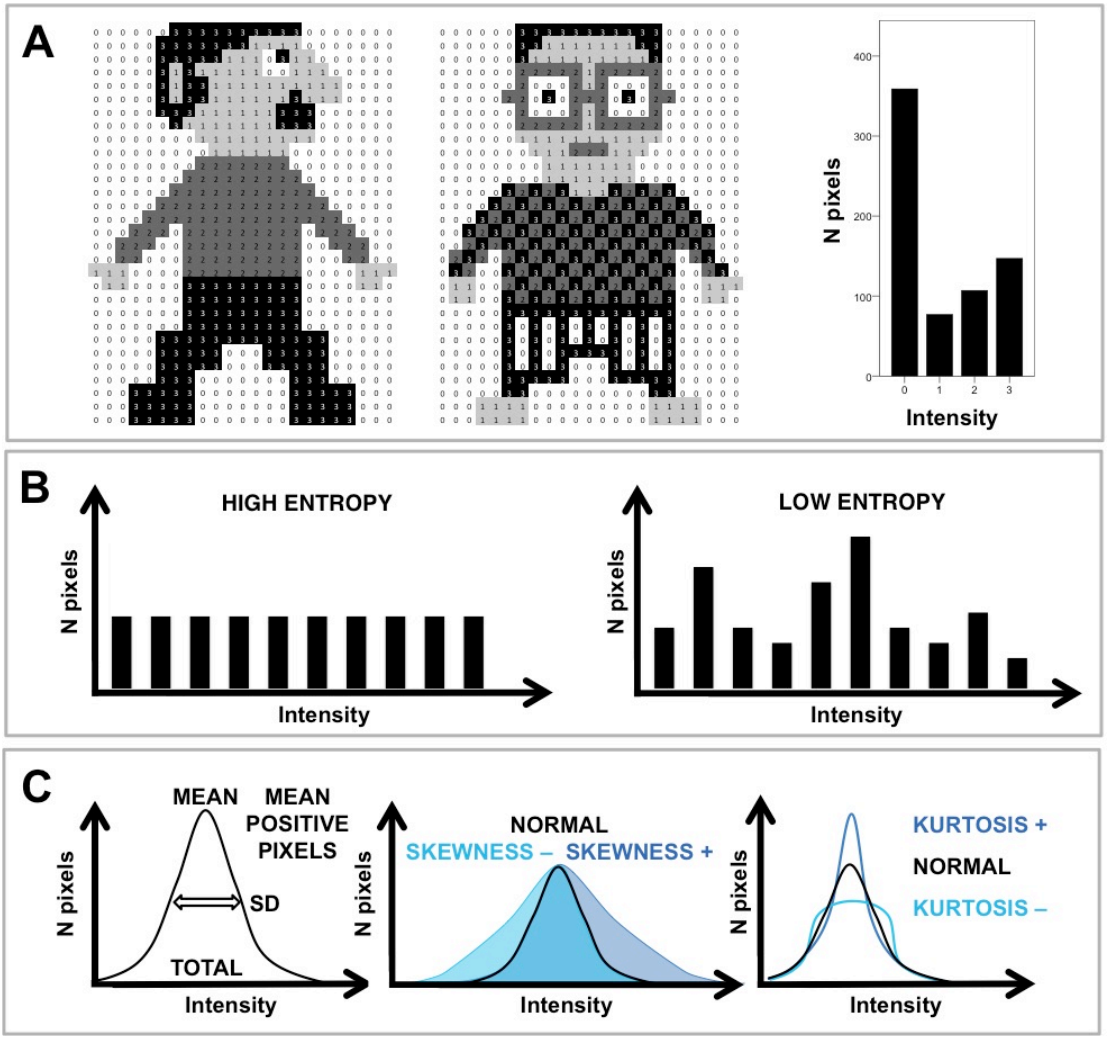

Supplemental Table 1. The variable Entropy is a logarithmic function of the area of the ROI in malignant and healthy tissues

Supplemental Table 1 legend. Logarithmic regression shows that the value of the variable Entropy is a function of the area of the ROI in every type of tissues (synchronous metastases, psoas muscle and aorta) in all SSFs. A similar equation is found in all cases. The high p-value (p<0.001) and R^2 show the prediction strength of this logarithmic model.

					p-value	R^2	F
**SM#1**	**Entropy SSF2 =**	3.07	+ 0.28	x ln(area of the ROI SSF2)	P<0.001	0.53	169
	**Entropy SSF3 =**	2.83	+ 0.29	x ln(area of the ROI SSF3)	P<0.001	0.51	154
	**Entropy SSF4 =**	1.89	+ 0.42	x ln(area of the ROI SSF4)	P<0.001	0.64	265
	**Entropy SSF5 =**	1.90	+ 0.40	x ln(area of the ROI SSF5)	P<0.001	0.65	262
	**Entropy SSF6 =**	1.19	+ 0.51	x ln(area of the ROI SSF6)	P<0.001	0.75	403
**SM#2**	**Entropy SSF2 =**	2.18	+ 0.42	x ln(area of the ROI SSF2)	P<0.001	0.74	317
	**Entropy SSF3 =**	1.78	+ 0.46	x ln(area of the ROI SSF3)	P<0.001	0.77	368
	**Entropy SSF4 =**	1.22	+ 0.54	x ln(area of the ROI SSF4)	P<0.001	0.84	535
	**Entropy SSF5 =**	1.23	+ 0.52	x ln(area of the ROI SSF5)	P<0.001	0.81	394
	**Entropy SSF6 =**	1.09	+ 0.54	x ln(area of the ROI SSF6)	P<0.001	0.79	307
**Muscle**	**Entropy SSF2 =**	2.92	+ 0.31	x ln(area of the ROI SSF2)	P<0.001	0.30	62
	**Entropy SSF3 =**	2.62	+ 0.31	x ln(area of the ROI SSF3)	P<0.001	0.36	79
	**Entropy SSF4 =**	1.54	+ 0.46	x ln(area of the ROI SSF4)	P<0.001	0.66	270
	**Entropy SSF5 =**	0.64	+ 0.60	x ln(area of the ROI SSF5)	P<0.001	0.82	611
	**Entropy SSF6 =**	0.55	+ 0.60	x ln(area of the ROI SSF6)	P<0.001	0.84	626
**Aorta**	**Entropy SSF2 =**	3.33	+ 0.21	x ln(area of the ROI SSF2)	P<0.001	0.40	98
	**Entropy SSF3 =**	2.94	+ 0.23	x ln(area of the ROI SSF3)	P<0.001	0.46	124
	**Entropy SSF4 =**	2.38	+ 0.30	x ln(area of the ROI SSF4)	P<0.001	0.59	207
	**Entropy SSF5 =**	1.51	+ 0.42	x ln(area of the ROI SSF5)	P<0.001	0.67	296
	**Entropy SSF6 =**	1.96	+ 0.35	x ln(area of the ROI SSF6)	P<0.001	0.59	194

Supplemental Table 2. Example of quality criteria for the implementation of imaging biomarkers into the clinics

Supplemental Table 1 legend. Example of quality criteria that might be used in further studies. Imaging biomarkers were measured in 112 patients in two synchronous metastases, psoas muscle and aorta (n=448).

* p <0.05

	ENTROPY	MEAN	SD	MPP	SKEWNESS	KURTOSIS
**Redundancy**<br>The biomarker is strongly correlated with another one (r>0.7)	SD		MPP, Ent.	SD		
**Recognition of the similarity between synchronous metastases**<br>Spearman's rho coefficient between SM#1 vs. SM#2 (n=112 pairs)	.52*	.68*	.43*	.43*	.24	-.02
**Volume-dependence**<br>Spearman's rho coefficient with the area of the ROI (n=448)	.75*	-.06*	.21*	.08*	.04*	.52*
**Stability across spatial scale filters (SSF)**<br>Spearman's rho coefficient between all SSF (n=448)	.83*	.83*	.73*	.73*	.58*	.48*

Supplemental Table 3. Stability of imaging biomarkers across spatial scale filters within the same ROI of the same lesions

Supplemental Table 3 legend. The values of imaging features are compared according to different SSF in 448 ROI (112 patients: SM#1, SM#2, psoas muscle and aorta). We see that Skewness and Kurtosis are volatile parameters. Spearman's Rho correlation coefficient is significant ($p<0.05$) in all cases.

**MEAN**	SSF2	SSF3	SSF4	SSF5
SSF3	.89			
SSF4	.78	.94		
SSF5	.69	.88	.96	
SSF6	.62	.80	.87	.94

**SKEWNESS**	SSF2	SSF3	SSF4	SSF5
SSF3	.69			
SSF4	.44	.82		
SSF5	.27	.60	.85	
SSF6	.23	.45	.65	.85

**SD**	SSF2	SSF3	SSF4	SSF5
SSF3	.85			
SSF4	.68	.91		
SSF5	.55	.77	.93	
SSF6	.38	.55	.75	.88

**KURTOSIS**	SSF2	SSF3	SSF4	SSF5
SSF3	.52			
SSF4	.33	.73		
SSF5	.23	.49	.80	
SSF6	.24	.30	.50	.75

**ENTROPY**	SSF2	SSF3	SSF4	SSF5
SSF3	.92			
SSF4	.82	.93		
SSF5	.71	.83	.95	
SSF6	.61	.71	.87	.95

**ROI AREA**	SSF2	SSF3	SSF4	SSF5
SSF3	.99			
SSF4	.96	.97		
SSF5	.90	.94	.98	
SSF6	.83	.88	.95	.97

**MPP**	SSF2	SSF3	SSF4	SSF5
SSF3	.83			
SSF4	.68	.89		
SSF5	.51	.73	.92	
SSF6	.40	.60	.82	.95
